# A Study of Alterations in DNA Epigenetic Modifications (5mC and 5hmC) and Gene Expression Influenced by Simulated Microgravity in Human Lymphoblastoid Cells

**DOI:** 10.1371/journal.pone.0147514

**Published:** 2016-01-28

**Authors:** Basudev Chowdhury, Arun Seetharam, Zhiping Wang, Yunlong Liu, Amy C. Lossie, Jyothi Thimmapuram, Joseph Irudayaraj

**Affiliations:** 1 Department of Biological Sciences, Purdue University, West Lafayette, IN, 47907, United States of America; 2 Bindley Biosciences Center, Discovery Park, Purdue University, West Lafayette IN, 47907, United States of America; 3 Department of Medical and Molecular Genetics, Indiana University School of Medicine Indianapolis, Indianapolis, IN, 46202, United States of America; 4 Center for Computational Biology and Bioinformatics, Indiana University School of Medicine Indianapolis, Indianapolis, IN, 46202, United States of America; 5 Bioinformatics Core, Purdue University, West Lafayette, IN, 47907, United States of America; 6 Department of Animal Sciences, Purdue University, West Lafayette, IN, 47907, United States of America; 7 Department of Agriculture and Biological Engineering, Purdue University, West Lafayette, IN, 47907, United States of America; Texas Tech University, UNITED STATES

## Abstract

Cells alter their gene expression in response to exposure to various environmental changes. Epigenetic mechanisms such as DNA methylation are believed to regulate the alterations in gene expression patterns. *In vitro* and *in vivo* studies have documented changes in cellular proliferation, cytoskeletal remodeling, signal transduction, bone mineralization and immune deficiency under the influence of microgravity conditions experienced in space. However microgravity induced changes in the epigenome have not been well characterized. In this study we have used Next-generation Sequencing (NGS) to profile ground-based “simulated” microgravity induced changes on DNA methylation (5-methylcytosine or 5mC), hydroxymethylation (5-hydroxymethylcytosine or 5hmC), and simultaneous gene expression in cultured human lymphoblastoid cells. Our results indicate that simulated microgravity induced alterations in the methylome (~60% of the differentially methylated regions or DMRs are hypomethylated and ~92% of the differentially hydroxymethylated regions or DHMRs are hyperhydroxymethylated). Simulated microgravity also induced differential expression in 370 transcripts that were associated with crucial biological processes such as oxidative stress response, carbohydrate metabolism and regulation of transcription. While we were not able to obtain any global trend correlating the changes of methylation/ hydroxylation with gene expression, we have been able to profile the simulated microgravity induced changes of 5mC over some of the differentially expressed genes that includes five genes undergoing differential methylation over their promoters and twenty five genes undergoing differential methylation over their gene-bodies. To the best of our knowledge, this is the first NGS-based study to profile epigenomic patterns induced by short time exposure of simulated microgravity and we believe that our findings can be a valuable resource for future explorations.

## Introduction

During space flight, astronauts are exposed to powerful environmental assaults such as microgravity, cosmic radiation and magnetic fields that have the potential to impinge upon cellular ontogeny through epigenetic modifications [1]. Throughout the evolutionary history, gravity has been a constant factor in defining the architecture and morphology of living beings [2]. Hence a broader understanding of gravity’s influence on biological functions is important for an accurate evaluation of risks associated with the health of astronauts in spaceflights and should be of enormous interest to the scientific community. The effects of microgravity in altering gene expression have been documented in mammalian cells [3, 4] and other model organisms, such as yeast and bacteria [5–7]. Microgravity associated pathological alterations include reduction in bone mass and calcium concentrations [8], alterations in hormonal levels [9], impairment of immunocompetence [10] and apoptosis signaling [11]. Studies of human lymphoblast and lymphoblast cell cultures following periods of simulated microgravity have demonstrated alterations in metabolic processes and DNA repair pathways which could in turn signify an increased susceptibility to malignancy [12, 13]. Collectively, these studies indicate exposure to microgravity during space flight alters gene expression patterns and subsequently cellular physiology.

DNA methylation is regarded as a major epigenetic mechanism and play key roles in regulating cellular processes in living organisms [14, 15]. Biochemically, DNA methylation refers to the addition of a methyl group (CH_3_) to the 5’ carbon on the pyrimidine ring of cytosine nucleotides (commonly abbreviated as 5mC). Aberrations in genome-wide 5mC patterns are widely prevalent in cancer and other diseases [14, 16–18]. Traditionally DNA methylation marks have been associated with “transcriptionally silent” genes, however the revelations of global methylation studies facilitated by recent advances in Next Generation Sequencing (NGS) tools have established that the role of 5mC in regulating gene expression is complex, varies according to the genomic context and warrants extensive explorations [19–25]. Discovered in 2009, DNA hydroxymethylation (5hmC) is a relatively new epigenetic modification occurring on Cytosine [26, 27] generated by Ten-Eleven Translocation (TET) protein- mediated oxidative catalysis of 5mC [26]. Though, potential roles of 5hmC at promoter and gene bodies are not clearly understood, it is shown to play some role in maintaining and/or promoting gene expression [14, 16–18, 28]. Microgravity induced alteration in DNA methylation patterns have been reported previously [29–31] but the effect of microgravity on 5hmC is virtually unknown. During the time period this study was being conducted there were no reports of a NGS based study documenting the effects of microgravity on the epigenomic landscape.

**Fig 1:**
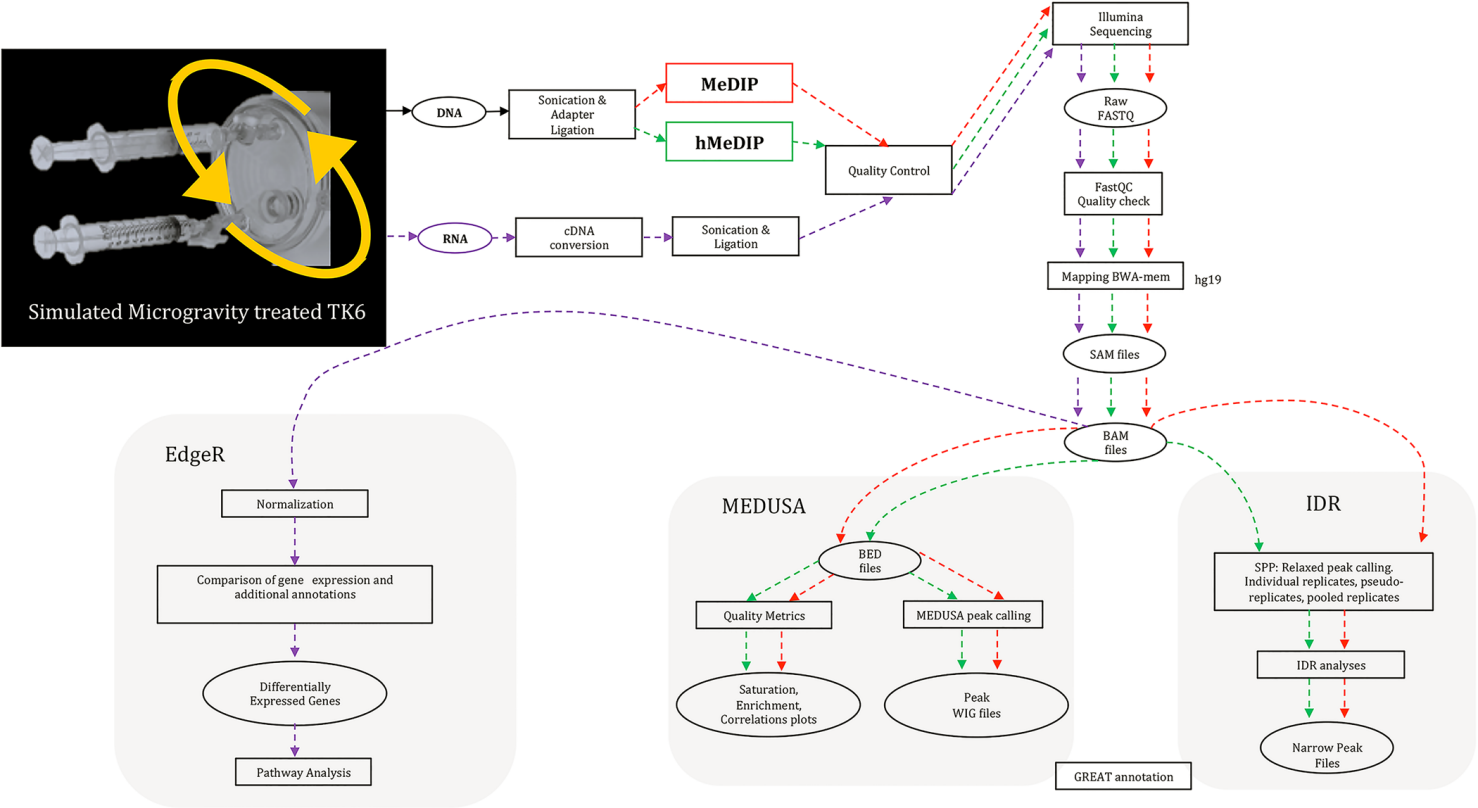
Schematic illustration of the bioinformatics pipeline for MeDIP-seq, hMeDIP-seq and RNA-seq analysis used in our study to understand the DNA methylation and hydroxymethylation and gene expression patterns induced by simulated microgravity. All steps were done in parallel in TK6 subjected to “simulated” microgravity and static controls under the influence of Earth’s gravitational force.

The goal of our study was to profile genome-wide effects of “simulated” microgravity on 5mC, 5hmC and gene expression patterns employing Next Generation Sequencing (**Fig 1**). The TK6 lymphoblastoid cell line, derived from T cell blast crisis of a patient with chronic myelogeneous leukemia [32], served as our model organism. TK6 cells are well characterized and have been extensively used as a substitute for peripheral blood lymphocytes for immunological and epidemiological studies [13]. The limited availability of biological specimen subjected to conditions of microgravity in spaceflights makes ground based “simulated” microgravity studies critical in determining thresholds and thorough testing of the model organism before conducting the experiments during space missions [33]. A High Aspect Ratio Vessel (HARV) based rotary cell culture system (initially developed by NASA) was used in our study to “simulate” microgravity in the TK6 cells as has been described previously [34] and compared to a control static cell-culture system under the influence of earth’s gravity. While assessing the merits of ground based “simulation” studies, it has to be appreciated that the effects of gravity cannot be completely negated but reduced to near zero to achieve a state of “functional near weightlessness” [33].

## Materials and Methods

### Cell culture

TK6 human lymphoblastoid cells (ATCC, Manassas, VA) were maintained in the log phase of cell growth by culturing in RPMI-1640 (Life Technologies, Grand Island, NY) medium supplemented with 10% Fetal Bovine Serum (Atlanta Biologicals, Flowery Branch, GA) and 1% Penicillin/Streptomycin (Life Technologies, Grand Island, NY) at 37°C in 5% CO_2_ and 95% air. For ground-based simulation of microgravity, HARV Rotary Cell Culture System (Synthecon, Houston, TX) was used. Actively growing TK6 cells were seeded in the bioreactor at 2 _X_ 10^5^ cells/ml and rotated at 12 rpm/min. In parallel, cells (at the same cellular density i.e. 2 _X_ 10^5^ cells/ml) were maintained in bioreactors in normal gravity (static) condition as controls. The bioreactors were maintained in an incubator at 37°C, with 5% CO_2_ and 95% air for 48 hours.

### DNA isolation, sonication and adapter ligation

Genomic DNA was isolated from the TK6 cells cultured under microgravity and control static conditions using the DNeasy Blood &Tissue kit (Qiagen Inc., Valencia, CA) following manufacturer’s instructions. 2.5 μg of genomic DNA from each sample was sheared using Covaris S2 Device (Covaris Inc., Woburn, MA). Sheared DNA was purified by binding to AmPure beads (Beckman Coulter Inc.) and End-repair performed by incubating sonicated DNA and End repair solution (New England Biolabs Inc., Ipswich, MA) as per manufacturer’s specifications. A-tailing was obtained by incubating the end repaired DNA with dA-tailing mix (New England Biolabs Inc., Ipswich, MA) at 37°C for 30 minutes. At this stage, to facilitate multiplexing each sample was equally divided in two parts (one half for MeDIP and the other half for hMeDIP respectively). Blunt end ligation was performed by incubating the A-tailed DNA samples (1 μg) with unmethylated versions of adapters (IDT Inc., Coralville, IA) based on sequences of the methylated Truseq adapters (Illumina Inc., San Diego, CA) for multiplexing. Thus 8 libraries were prepared as described above. Samples were assayed by qPCR in duplicate and standard curve constructed to establish the molarities of the libraries.

### MeDIP-seq /hMeDIP-seq

MeDIP and hMeDIP were performed using the methylated/hydroxymethylated DNA enrichment kits (Diagenode Inc., Denville, NJ) following the manufacturer’s protocol. Briefly, to 1.2 μg of adapter ligated sonicated genomic DNA, three DNA controls (known sequences bearing unmethylated, methylated or hydroxymethylated Cytosines respectively to assess the efficiency of immunoprecipitation reactions) were spiked-in. The concentration of genomic DNA was adjusted to incorporate the addition of the adapter sequences, preserving the appropriate molar ratio between the genomic DNA and anti-5mC/anti-5hmC antibody during MeDIP/hMeDIP as described by Butcher *et al*. [35]:

$$[conc.gDNA]=\frac{[conc.adapter\;ligated\;gDNA]\;*\;bpsonicated\;gDNA}{bpsonicated\;gDNA+bpadapter\;DNA}$$

Where, $Conc(adjusted)=\frac{Conc(ligated)}{\left\{\frac{bp(sample)+bp(adapter}{bp(sample)}\right\}}$

**conc.***gDNA ➔* adjusted genomic DNA concentration in the adapter ligated libraries,

**conc.***adapter ligated gDNA ➔* concentration of the adapter ligated gDNA libraries,

**bp***sonicated gDNA ➔* average size of the pre-ligation sonicated gDNA and

**bp***adapter DNA ➔* average size of the adapters

After incubation at 95°C to denature the double stranded DNA, immunoprecipitation was performed by incubation with monoclonal antibody directed against 5mC/5hmC (Diagenode Inc., Denville, NJ) and secondary antibody with magnetic bead conjugates (Diagenode Inc., Denville, NJ) overnight at 4°C while being spun continuously at 40 rpm. The captured 5mC/5hmC bearing DNA fragments were separated from the others by magnetic pulldown. After repeated cleanups, the captured DNA was isolated from the magnetic beads bearing antibody using the IPure kit (Diagenode Inc., Denville, NJ). The enrichment of 5mC/5hmC bearing DNA was assessed by performing qPCR on the pre and post immunoprecipitated samples. As a control, an identical immunoprecipitation reaction with mouse IgG instead of monoclonal 5mC/5hmC antibody was performed. The methylated/hydroxymethylated DNA immunoprecipitated libraries were amplified by PCR and submitted to the Purdue Genomics Core Facility for high-throughput sequencing by Hi Seq 2000 (Illumina Inc., San Diego, CA).

### MeDIP-seq and hMeDIP-seq data processing

FastQC v 0.10.1 [36] was used to assess the quality of the reads and to generate graphical representations of numerous quality metrics (per base sequence quality, GC content and sequence duplication/size distribution levels). The reads were aligned to human reference genome hg19 using BWA v 0.6.2 [37], with default parameters and a maximum insert size of 400 bp. The resulting SAM files were converted to BAM format and sorted using Samtools v0.1.18 [38] as illustrated in (**Fig 1**). PERL script from the MeDUSA package [39] was used to convert the BAM files to BED format. Since the MEDIPS v1.0 [40] package requires only selective fields as input, the BED format was then reduced to four fields using the UNIX cut option. The MeDUSA pipeline utilizes the Bioconductor package MEDIPS v1.0 and custom R scripts to calculate quality metrics for the MeDIP-seq data were designed. The data was normalized for the size of the sequence libraries by calculating reads per million (RPM) in tiled windows across the genome. Wig files obtained for the normalized read depth following alignment and filtering were presented as RPM. Quality check on the MeDIP-seq data was also performed by calculating CpG enrichment values, saturation plots and coverage plots. Genome-wide correlations between the replicates were performed as a quality check for consistency among the replicates using QCSeqs from the Useq package (v8.40) [41] using a window size of 500 bp, increasing in 250 bp increments and a minimum number of 5 reads in a window.

### Identification of DMRs and DHMRs

Peak calling software SPP v1.10 [42], was used to call peaks and rank them based on significance of enrichment (p-values and false discovery rates). IDR (Irreproducible Discovery Rate) framework was used to measure experiment quality in terms of reproducibility [43] and to select the reproducible, consistent peaks (overlapped significant peaks from both replicates) determined based on IDR values. The threshold of 0.05 IDR was used for truncating the peak list as suggested by the developers. The differentially methylated/hydroxymethylated regions identified by IDR analyses were then annotated with their chromosomal locations and feature types for further biological interpretation using custom Perl scripts of MeDUSA package, BEDTools [44] and feature annotation files (GFF files from UCSC) as illustrated in **Fig 1**. Further annotation (plots for enrichment of 5mC/5hmC) was done using CEAS (v1.02) [45] and proximity of the peaks to the TSS was determined using PeakAnalyzer [46]. The FDR value of 0.05 was used as cut-off for all peak association studies. The complete MeDIP-seq and hmeDIP-seq data was submitted to NCBI GEO (GSE65944) and available in the database http://www.ncbi.nlm.nih.gov/geo/query/acc.cgi?acc=GSE65944).

### RNA-seq

Total RNA was extracted from cells subjected to simulated microgravity and static control using RNA-STAT-60 (Tel-Test,Inc., Friendswood, TX) using the manufacturer’s instructions. Briefly, 1ml of RNA-STAT solution was added per 10^6^ cells and homogenized for 5 minutes over ice. 1ml of chloroform was added, contents shaken vigorously and centrifuged at 12,000g at 4°C for 15 minutes. The aqueous solution was transferred to corex tube (Corning Inc., Lowell, MA) and 0.8 ml isopropanol added. After incubation of 10 minutes, the contents were centrifuged at 12,000g for another 10 minutes to precipitate the RNA. The RNA pellet was washed with 75% ethanol and centrifuged at 7,500g for 5 minutes at 4°C. The ethanol was aspirated and the RNA pellet dried. The RNA pellet was finally resuspended in DEPC water and submitted to the Purdue Genomic Center for conversion into cDNA, sonication, adapter ligation and sequencing as described previously. The reads (fastq files) were aligned to human reference genome hg19 using Tophat v2.1.0 [47], with default parameters and known transcriptome as illustrated in **Fig 1**. Alignment results were filtered by Bamutils v0.5.0 [48] to remove reads with multiple mappings. Statistics data of the resulting alignment files were created using Samtools v0.1.18 [49] and Bamutils v0.5.0. The counts of aligned reads mapping to known genes were calculated using bamutils v0.5.0. EdgeR v2.11 [50] was used to compute the differentially expressed genes. Pathway analysis on the set of differentially expressed genes was done using the GeneCodis3 software designed at the Complutense University of Madrid [51].The complete RNA-seq data was submitted to NCBI GEO (GSE65944) and available in the database (http://www.ncbi.nlm.nih.gov/geo/query/acc.cgi?acc=GSE65944).

## Results

### Effect of simulated microgravity on cell growth and viability

The effect of simulated microgravity on cell growth and viability 48 hours after the cells were seeded in bioreactors in either the rotating or static condition was determined using Trypan Blue staining method by Automated Cell Counter (Nexcelom Bioscience LLC., Lawrence, MA) in **S1 Fig**. No significant differences in the percentage of viable cells between the two cell culture conditions after 48 hours was observed. Specifically, 95.1 ± 2.12% of the cells subjected to simulated microgravity were viable, while 91.1 ± 3.54% of the static control cells were determined to be viable. The average cellular diameter (μm) was determined to be 12.6 ± 0.42 and 12.8 ± 0.28 in TK6 cells subjected to microgravity and control respectively. Similar cellular growth rates between the rotating and static culture conditions facilitated ruling out the possibility of cell growth being the major contributor to the changes in the methylation and gene expression patterns.

### Changes in 5mC profile following simulated microgravity

We applied MeDIP coupled with high-throughput sequencing to identify the differences in the genome-wide patterns of 5mC upon simulated microgravity on TK6 cells. 2.8x10^8^ and 1.8x10^8^ reads were obtained during MeDIP-seq from TK6 cells subjected to static and simulated microgravity respectively and more than 90% of these reads aligned to the human genome GRCh37/hg19, 2009 Assembly (**S1 Table and S2 Fig**). Quality assessment generated by FastQC [36] showed satisfactory sequence quality for all measures except for GC content. As GC rich regions of the genome are enriched in MeDIP-seq datasets, this result was not unexpected. The depth of sequencing for MeDIP-seq samples ranged from 2.8X to 6.1X **(S1 Table).** Cross-correlation analysis was performed as per the ENCODE consortium guidelines [42, 52, 53] and all the samples displayed Normalized Strand Correlation (NSC) and Relative Strand Correlation (RSC) values (**S1 Table**) characteristic of “high-quality data sets”. The similarity between the replicates was evident as hierarchical dendrogram displayed distinct clustering of biological replicates in two groups (**S3 Fig**) and sequence coverage analyses displayed that MeDIP-seq reads generated from the samples covered similar number of bases of the reference genome **(S4 Fig).**

**Fig 2:**
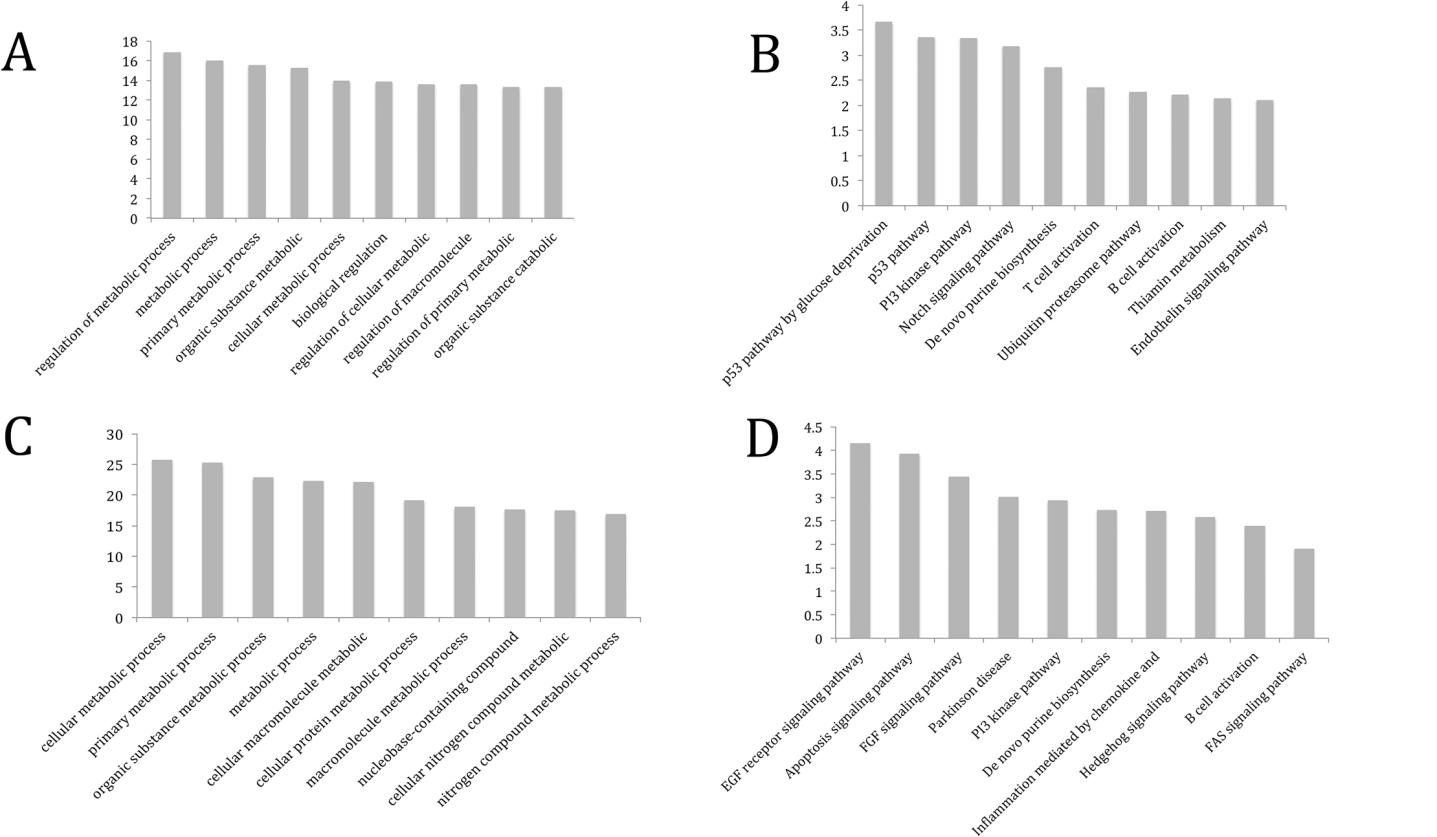
Pathways illustrating the network of genomic loci involved with (A & B) Regions undergoing increase in 5mC content and (C & D) decrease in 5mC contents, upon simulated microgravity.

Differentially methylated region (DMRs) were defined as genomic regions in TK6 cells under simulated microgravity that showed alteration in methylation (either increase or decrease) compared to TK6 cells under static conditions. 3204 DMRs (**S2 Table & S3 Table**) were detected using the IDR pipeline having an IDR cutoff value of 0.05 or less. Of the total DMRs, 1286 (40.14%) were associated with hypermethylation (gain-of-5mC) (**S2 Table)** and 1918 (59.86%) with hypomethylation (loss-of-5mC) (**S3 Table)** upon simulated microgravity respectively. The DMRs were further analyzed to determine the overlap of DMR regions with different genomic features by the methylome analysis pipeline described in details by Wilson et al. [39]. Functional genomic distribution analyses indicated that 969 and 1381 genes associated with DMRs have undergone gain-of-5mC and loss-of-5mC respectively (**Table 1**). Also, 105 hypermethylated and 193 hypomethylated DMRs were observed around -1500 to 1500 bps of Transcription Start Sites (TSS) as demonstrated in **Tables 2 & 3**. The distribution of the genomic repeat sequences (LINE, SINE and LTR) located within the DMRs has been represented in **Table 1**. Metadata describing features such as genes, transcripts, Pseudogene, non-coding RNA and other regulatory features present on each DMR has been included in **S2 Table & S3 Table**. Investigation of annotations from 20 different ontologies from genomic coordinates of DMRs was generated by utilizing Stanford University’s Genomic Regions Enrichment of Annotations Tool (GREAT) version 3.0.0 [54] and included in **S4 Table & S5 Table.** Gain-of-5mC DMRs induced by simulated microgravity were found to enrich GO Biological Processes like regulation of metabolic process (GO: 0019222), primary metabolic process (GO: 0044238) and cellular metabolic process (GO: 0044237) **(Fig 2A)**. PANTHER Pathway Analysis implicated genes involved in p53 pathway (P00059), PI3 kinase pathway (P00048), T cell activation (P00053) and B cell activation (P00010) to be associated with hypermethylated DMRs **(Fig 2B)**. On the other hand, loss-of-5mC DMRs were observed to enrich GO Biological Processes like cellular metabolic process (GO: 0044237) and primary metabolic process (GO: 0044238) **(Fig 2C**). PANTHER Pathway Analysis, revealed that these hypomethylated DMRs were associated with genes involved in EGF receptor signaling (P00018), Apoptosis signaling (P00006) and FGF signaling (P00021) pathways among others **(Fig 2D)**.

**Table 1:** Genome annotation Summary. The number of genomic features such as CpG islands, CpG shores, ENSEMBL Genes and DNA Repeats (LINE, SINE and LTR) associated with regions undergoing gain-of-5mC/5hmC and loss-of-5mC/5hmC DMRs or DHMRs in TK6 cells cultured under simulated microgravity compared to static condition.

Features	DMR		DHMR	
	Gain-of-5mC	Loss-of-5mC	Gain-of-5hmC	Loss-of-5hmC
**CpGI**	23	69	1	0
**CpG**	127	277	5	1
**Gene**	969	1381	86	7
**LINE**	421	521	47	1
**SINE**	944	1973	18	11
**LTR**	157	227	28	0

**Table 2:** List of hypermethylated DMRs located within +/- 1500 of Transcription Start Sites of genes. Columns display the genomic coordinates of DMRs, Gene Symbol of the corresponding gene, the description of the genome and the exact distance in bp.

DMR (Chr:Start-End)	Gene Symbol	Description	Distance
2:74407290–74407690	MOB1A	MOB kinase activator 1A	-1495
1:32620788–32621188	KPNA6	karyopherin alpha 6 (importin alpha 7)	-1475
8:48919307–48919707	UBE2V2	ubiquitin-conjugating enzyme E2 variant 2	-1453
20:10644375–10644775	JAG1	jagged 1	-1421
19:8526792–8527192	HNRNPM	heterogeneous nuclear ribonucleoprotein M	-1389
10:103579850–103580250	MGEA5	meningioma expressed antigen 5 (hyaluronidase)	-1354
1:176177694–176178094	RFWD2	ring finger and WD repeat domain 2, E3 ubiquitin protein ligase	-1265
8:66547493–66547893	ARMC1	armadillo repeat containing 1	-1251
17:73976545–73976945	ACOX1	acyl-CoA oxidase 1, palmitoyl	-1230
5:137912163–137912563	HSPA9	heat shock 70kDa protein 9 (mortalin)	-1230
1:9710401–9710801	PIK3CD	phosphatidylinositol-4,5-bisphosphate 3-kinase, catalytic subunit delta	-1189
17:76820294–76820694	USP36	ubiquitin specific peptidase 36	-1163
7:144108260–144108660	NOBOX	NOBOX oogenesis homeobox	-1140
1:179333515–179333915	AXDND1	axonemal dynein light chain domain containing 1	-1140
7:150781644–150782044	AGAP3	ArfGAP with GTPase domain, ankyrin repeat and PH domain 3	-1110
3:27765104–27765504	EOMES	eomesodermin	-1098
1:27096449–27096849	ARID1A	AT rich interactive domain 1A (SWI-like)	-1071
17:37608338–37608738	MED1	mediator complex subunit 1	-999
4:94748859–94749259	ATOH1	atonal homolog 1 (Drosophila)	-983
10:111968848–111969248	MXI1	MAX interactor 1, dimerization protein	-941
20:35488994–35489394	SOGA1	suppressor of glucose, autophagy associated 1	-918
22:51067323–51067723	ARSA	arylsulfatase A	-916
10:112630503–112630903	PDCD4	programmed cell death 4 (neoplastic transformation inhibitor)	-862
16:75468037–75468437	CFDP1	craniofacial development protein 1	-854
17:4235771–4236171	UBE2G1	ubiquitin-conjugating enzyme E2G 1	-753
17:47492799–47493199	PHB	prohibitin	-753
3:99978894–99979294	TBC1D23	TBC1 domain family, member 23	-750
17:33469869–33470269	NLE1	notchless homolog 1 (Drosophila)	-735
17:65026584–65026984	AC005544.1	Uncharacterized protein	-725
2:88895888–88896288	EIF2AK3	eukaryotic translation initiation factor 2-alpha kinase 3	-713
1:150265379–150265779	MRPS21	mitochondrial ribosomal protein S21	-710
4:37827346–37827746	PGM2	phosphoglucomutase 2	-709
20:42573450–42573850	TOX2	TOX high mobility group box family member 2	-695
1:151738242–151738642	OAZ3	ornithine decarboxylase antizyme 3	-689
11:33277352–33277752	HIPK3	homeodomain interacting protein kinase 3	-666
15:60691398–60691798	ANXA2	annexin A2	-657
17:3716988–3717388	C17orf85	chromosome 17 open reading frame 85	-644
10:8095826–8096226	GATA3	GATA binding protein 3	-630
9:123295232–123295632	CDK5RAP2	CDK5 regulatory subunit associated protein 2	-599
7:48019521–48019921	HUS1	HUS1 checkpoint homolog (S. pombe)	-571
12:120109292–120109692	PRKAB1	protein kinase, AMP-activated, beta 1 non-catalytic subunit	-557
15:43637360–43637760	ADAL	adenosine deaminase-like	-545
5:122180427–122180827	SNX24	sorting nexin 24	-517
3:113676612–113677012	ZDHHC23	zinc finger, DHHC-type containing 23	-489
19:58400668–58401068	ZNF814	zinc finger protein 814	-463
14:93184061–93184461	LGMN	legumain	-457
20:45280352–45280752	SLC13A3	solute carrier family 13 (sodium-dependent dicarboxylate transporter), member 3	-454
2:8977951–8978351	KIDINS220	kinase D-interacting substrate, 220kDa	-391
20:30639472–30639872	HCK	hemopoietic cell kinase	-319
5:67521976–67522376	PIK3R1	phosphoinositide-3-kinase, regulatory subunit 1 (alpha)	-286
16:25027042–25027442	ARHGAP17	Rho GTPase activating protein 17	-255
19:11306249–11306649	KANK2	KN motif and ankyrin repeat domains 2	-88
12:110783800–110784200	ATP2A2	ATPase, Ca++ transporting, cardiac muscle, slow twitch 2	-6
13:78493694–78494094	EDNRB	endothelin receptor type B	9
13:111839015–111839415	ARHGEF7	Rho guanine nucleotide exchange factor (GEF) 7	14
3:180633094–180633494	FXR1	fragile X mental retardation, autosomal homolog 1	46
12:122457270–122457670	BCL7A	B-cell CLL/lymphoma 7A	142
14:95569392–95569792	DICER1	dicer 1, ribonuclease type III	175
3:64253200–64253600	PRICKLE2	prickle homolog 2 (Drosophila)	255
19:45445601–45446001	APOC4	apolipoprotein C-IV	270
13:114549584–114549984	GAS6	growth arrest-specific 6	272
5:68470171–68470571	CCNB1	cyclin B1	287
4:89079332–89079732	ABCG2	ATP-binding cassette, sub-family G (WHITE), member 2	291
22:41257564–41257964	DNAJB7	DnaJ (Hsp40) homolog, subfamily B, member 7	366
15:83676793–83677193	C15orf40	chromosome 15 open reading frame 40	375
13:113301551–113301951	C13orf35	chromosome 13 open reading frame 35	393
9:130340673–130341073	FAM129B	family with sequence similarity 129, member B	395
21:34804877–34805277	IFNGR2	interferon gamma receptor 2 (interferon gamma transducer 1)	451
12:70728471–70728871	CNOT2	CCR4-NOT transcription complex, subunit 2	456
4:80993052–80993452	ANTXR2	anthrax toxin receptor 2	465
19:58741108–58741508	ZNF544	zinc finger protein 544	474
11:85565301–85565701	AP000974.1	CDNA FLJ26432 fis, clone KDN01418; Uncharacterized protein	485
19:40831600–40832000	C19orf47	chromosome 19 open reading frame 47	530
5:137070955–137071355	KLHL3	kelch-like family member 3	549
19:10120383–10120783	COL5A3	collagen, type V, alpha 3	564
17:78389846–78390246	ENDOV	endonuclease V	577
3:101231200–101231600	SENP7	SUMO1/sentrin specific peptidase 7	628
13:28673851–28674251	FLT3	fms-related tyrosine kinase 3	656
18:32919753–32920153	ZNF24	zinc finger protein 24	665
2:202644765–202645165	ALS2	amyotrophic lateral sclerosis 2 (juvenile)	680
17:17183036–17183436	COPS3	COP9 constitutive photomorphogenic homolog subunit 3 (Arabidopsis)	778
19:12661227–12661627	ZNF564	zinc finger protein 564	821
8:107283104–107283504	OXR1	oxidation resistance 1	831
2:25390345–25390745	POMC	proopiomelanocortin	895
3:192959642–192960042	HRASLS	HRAS-like suppressor	928
19:54662449–54662849	LENG1	leukocyte receptor cluster (LRC) member 1	971
12:100595414–100595814	ACTR6	ARP6 actin-related protein 6 homolog (yeast)	985
13:27828691–27829091	RPL21	ribosomal protein L21	1049
12:57858360–57858760	GLI1	GLI family zinc finger 1	1085
17:74476687–74477087	RHBDF2	rhomboid 5 homolog 2 (Drosophila)	1089
19:38712475–38712875	DPF1	D4, zinc and double PHD fingers family 1	1138
19:53138925–53139325	ZNF83	zinc finger protein 83	1214
9:6644198–6644598	GLDC	glycine dehydrogenase (decarboxylating)	1252
3:172362558–172362958	AC007919.2	HCG1787166; PRO1163	1275
17:71230451–71230851	C17orf80	chromosome 17 open reading frame 80	1286
12:123875689–123876089	SETD8	SET domain containing (lysine methyltransferase) 8	1300
7:98479823–98480223	TRRAP	transformation/transcription domain-associated protein	1310
7:72396789–72397189	POM121	POM121 transmembrane nucleoporin	1329
4:187646331–187646731	FAT1	FAT tumor suppressor homolog 1 (Drosophila)	1345
2:216256316–216256716	FN1	fibronectin 1	1354
17:40654447–40654847	ATP6V0A1	ATPase, H+ transporting, lysosomal V0 subunit a1	1387
12:111857341–111857741	SH2B3	SH2B adaptor protein 3	1397
12:110925896–110926296	FAM216A	family with sequence similarity 216, member A	1400
2:53996217–53996617	CHAC2	ChaC, cation transport regulator homolog 2 (E. coli)	1488
2:947917–948317	SNTG2	syntrophin, gamma 2	1492

**Table 3:** List of hypomethylated DMRs located within +/- 1500 of Transcription Start Sites of genes. Columns display the genomic coordinates of DMRs, Gene Symbol of the corresponding gene, the description of the genome and the exact distance in base pairs.

DMR (Chr:Start-End)	Gene Symbol	Description	Distance
1:45958152–45958568	TESK2	testis-specific kinase 2	-1488
13:21138922–21139338	IFT88	intraflagellar transport 88 homolog (Chlamydomonas)	-1455
12:6831258–6831674	COPS7A	COP9 constitutive photomorphogenic homolog subunit 7A (Arabidopsis)	-1441
11:107990630–107991046	ACAT1	acetyl-CoA acetyltransferase 1	-1405
19:569701–570117	BSG	basigin (Ok blood group)	-1388
19:54664797–54665213	LENG1	leukocyte receptor cluster (LRC) member 1	-1385
14:50232737–50233153	KLHDC2	kelch domain containing 2	-1381
11:47289128–47289544	MADD	MAP-kinase activating death domain	-1376
17:33465350–33465766	NLE1	notchless homolog 1 (Drosophila)	-1372
2:72372691–72373107	CYP26B1	cytochrome P450, family 26, subfamily B, polypeptide 1	-1355
20:34543689–34544105	SCAND1	SCAN domain containing 1	-1349
8:76318743–76319159	HNF4G	hepatocyte nuclear factor 4, gamma	-1320
17:48946441–48946857	TOB1	transducer of ERBB2, 1	-1310
16:3931818–3932234	CREBBP	CREB binding protein	-1299
2:211306550–211306966	LANCL1	LanC lantibiotic synthetase component C-like 1 (bacterial)	-1289
1:47780891–47781307	STIL	SCL/TAL1 interrupting locus	-1280
17:16333898–16334314	TRPV2	transient receptor potential cation channel, subfamily V, member 2	-1263
3:12706770–12707186	RAF1	v-raf-1 murine leukemia viral oncogene homolog 1	-1253
19:49577242–49577658	KCNA7	potassium voltage-gated channel, shaker-related subfamily, member 7	-1252
5:175974933–175975349	CDHR2	cadherin-related family member 2	-1251
5:156363707–156364123	TIMD4	T-cell immunoglobulin and mucin domain containing 4	-1228
12:49111699–49112115	CCNT1	cyclin T1	-1226
17:79607945–79608361	TSPAN10	tetraspanin 10	-1196
1:25257327–25257743	RUNX3	runt-related transcription factor 3	-1167
12:110460055–110460471	ANKRD13A	ankyrin repeat domain 13A	-1161
22:43011912–43012328	POLDIP3	polymerase (DNA-directed), delta interacting protein 3	-1152
13:52981570–52981986	THSD1	thrombospondin, type I, domain containing 1	-1149
9:103203198–103203614	MSANTD3-TMEFF1	MSANTD3-TMEFF1 readthrough	-1147
9:123584680–123585096	PSMD5	proteasome (prosome, macropain) 26S subunit, non-ATPase, 5	-1144
19:10827435–10827851	DNM2	dynamin 2	-1112
1:32404887–32405303	PTP4A2	protein tyrosine phosphatase type IVA, member 2	-1107
2:203129141–203129557	NOP58	NOP58 ribonucleoprotein	-1090
12:53474061–53474477	SPRYD3	SPRY domain containing 3	-1065
4:87814423–87814839	C4orf36	chromosome 4 open reading frame 36	-1062
17:29157723–29158139	ATAD5	ATPase family, AAA domain containing 5	-1057
19:50029617–50030033	RCN3	reticulocalbin 3, EF-hand calcium binding domain	-1050
9:19150115–19150531	PLIN2	perilipin 2	-1047
7:138666899–138667315	KIAA1549	KIAA1549	-1043
11:66446181–66446597	RBM4B	RNA binding motif protein 4B	-997
16:88783445–88783861	PIEZO1	piezo-type mechanosensitive ion channel component 1	-968
5:176828391–176828807	PFN3	profilin 3	-962
6:35309180–35309596	PPARD	peroxisome proliferator-activated receptor delta	-947
3:52805678–52806094	NEK4	NIMA-related kinase 4	-921
14:52291822–52292238	GNG2	guanine nucleotide binding protein (G protein), gamma 2	-883
1:150292846–150293262	PRPF3	PRP3 pre-mRNA processing factor 3 homolog (S. cerevisiae)	-871
1:28560198–28560614	DNAJC8	DnaJ (Hsp40) homolog, subfamily C, member 8	-870
17:1626772–1627188	WDR81	WD repeat domain 81	-854
16:72137332–72137748	DHX38	DEAH (Asp-Glu-Ala-His) box polypeptide 38	-845
18:77961381–77961797	PARD6G	par-6 partitioning defective 6 homolog gamma (C. elegans)	-825
13:42621863–42622279	DGKH	diacylglycerol kinase, eta	-818
13:77461149–77461565	KCTD12	potassium channel tetramerisation domain containing 12	-817
22:43037207–43037623	ATP5L2	ATP synthase, H+ transporting, mitochondrial Fo complex, subunit G2	-808
15:42076825–42077241	AC073657.1		-804
5:170189356–170189772	GABRP	gamma-aminobutyric acid (GABA) A receptor, pi	-790
20:34000467–34000883	UQCC	ubiquinol-cytochrome c reductase complex chaperone	-731
9:99802448–99802864	CTSL2	cathepsin L2	-731
22:50766008–50766424	DENND6B	DENN/MADD domain containing 6B	-727
22:19279757–19280173	CLTCL1	clathrin, heavy chain-like 1	-726
1:154244054–154244470	HAX1	HCLS1 associated protein X-1	-725
9:125591423–125591839	PDCL	phosducin-like	-721
15:40401584–40402000	BMF	Bcl2 modifying factor	-699
12:48745657–48746073	ZNF641	zinc finger protein 641	-668
1:107598393–107598809	PRMT6	protein arginine methyltransferase 6	-666
5:134073321–134073737	CAMLG	calcium modulating ligand	-662
19:2256862–2257278	JSRP1	junctional sarcoplasmic reticulum protein 1	-654
4:17783579–17783995	FAM184B	family with sequence similarity 184, member B	-652
6:25965887–25966303	TRIM38	tripartite motif containing 38	-649
2:242186700–242187116	HDLBP	high density lipoprotein binding protein	-629
7:43909561–43909977	MRPS24	mitochondrial ribosomal protein S24	-613
20:44440411–44440827	UBE2C	ubiquitin-conjugating enzyme E2C	-596
8:99075748–99076164	C8orf47	chromosome 8 open reading frame 47	-583
8:19680112–19680528	INTS10	integrator complex subunit 10	-579
15:60885693–60886109	RORA	RAR-related orphan receptor A	-576
9:124856243–124856659	TTLL11	tubulin tyrosine ligase-like family, member 11	-566
12:123948281–123948697	SNRNP35	small nuclear ribonucleoprotein 35kDa (U11/U12)	-564
4:190861171–190861587	FRG1	FSHD region gene 1	-564
19:11450690–11451106	RAB3D	RAB3D, member RAS oncogene family	-554
10:126489606–126490022	FAM175B	family with sequence similarity 175, member B	-540
7:129691616–129692032	ZC3HC1	zinc finger, C3HC-type containing 1	-533
13:103458965–103459381	RP11-484I6.7	BIVM-ERCC5 protein	-531
22:30475426–30475842	HORMAD2	HORMA domain containing 2	-529
3:155463174–155463590	PLCH1	phospholipase C, eta 1	-526
19:39970330–39970746	TIMM50	translocase of inner mitochondrial membrane 50 homolog (S. cerevisiae)	-514
19:56347244–56347660	NLRP4	NLR family, pyrin domain containing 4	-492
1:26871647–26872063	RPS6KA1	ribosomal protein S6 kinase, 90kDa, polypeptide 1	-488
11:62555950–62556366	TMEM179B	transmembrane protein 179B	-483
19:2740422–2740838	SLC39A3	solute carrier family 39 (zinc transporter), member 3	-480
8:146176515–146176931	ZNF16	zinc finger protein 16	-449
2:175202383–175202799	AC018470.1	Uncharacterized protein FLJ46347	-440
3:141120576–141120992	ZBTB38	zinc finger and BTB domain containing 38	-407
1:27213196–27213612	GPN2	GPN-loop GTPase 2	-388
19:12946378–12946794	RTBDN	retbindin	-344
10:28032598–28033014	MKX	mohawk homeobox	-332
6:44923360–44923776	SUPT3H	suppressor of Ty 3 homolog (S. cerevisiae)	-321
21:34926838–34927254	SON	SON DNA binding protein	-309
20:4880379–4880795	SLC23A2	solute carrier family 23 (nucleobase transporters), member 2	-294
21:45078094–45078510	HSF2BP	heat shock transcription factor 2 binding protein	-277
13:50070769–50071185	PHF11	PHD finger protein 11	-272
16:22018485–22018901	C16orf52	chromosome 16 open reading frame 52	-266
11:17372825–17373241	NCR3LG1	natural killer cell cytotoxicity receptor 3 ligand 1	-240
20:36149179–36149595	NNAT	neuronatin	-230
14:96670598–96671014	BDKRB2	bradykinin receptor B2	-210
6:84419386–84419802	SNAP91	synaptosomal-associated protein, 91kDa	-184
17:57983959–57984375	RPS6KB1	ribosomal protein S6 kinase, 70kDa, polypeptide 1	-182
9:130660260–130660676	ST6GALNAC6	ST6 (alpha-N-acetyl-neuraminyl-2,3-beta-galactosyl-1,3)-N-acetylgalactosaminide alpha-2,6-sialyltransferase 6	-177
1:107683060–107683476	NTNG1	netrin G1	-174
18:2846660–2847076	EMILIN2	elastin microfibril interfacer 2	-160
17:73528230–73528646	LLGL2	lethal giant larvae homolog 2 (Drosophila)	-136
3:55521255–55521671	WNT5A	wingless-type MMTV integration site family, member 5A	-132
4:99578749–99579165	TSPAN5	tetraspanin 5	-114
17:72580766–72581182	C17orf77	chromosome 17 open reading frame 77	-83
17:73085977–73086393	SLC16A5	solute carrier family 16, member 5 (monocarboxylic acid transporter 6)	-72
11:62445317–62445733	UBXN1	UBX domain protein 1	-70
2:219906078–219906494	CCDC108	coiled-coil domain containing 108	-41
1:47697216–47697632	TAL1	T-cell acute lymphocytic leukemia 1	-37
17:73230571–73230987	NUP85	nucleoporin 85kDa	-20
4:111397002–111397418	ENPEP	glutamyl aminopeptidase (aminopeptidase A)	-19
19:11658471–11658887	CNN1	calponin 1, basic, smooth muscle	24
4:47839834–47840250	CORIN	corin, serine peptidase	47
15:55790255–55790671	DYX1C1	dyslexia susceptibility 1 candidate 1	83
21:34924435–34924851	SON	SON DNA binding protein	89
15:57025972–57026388	ZNF280D	zinc finger protein 280D	104
6:137815204–137815620	OLIG3	oligodendrocyte transcription factor 3	119
14:23652505–23652921	SLC7A8	solute carrier family 7 (amino acid transporter light chain, L system), member 8	136
12:31812613–31813029	METTL20	methyltransferase like 20	176
14:72400014–72400430	RGS6	regulator of G-protein signaling 6	273
2:98703769–98704185	VWA3B	von Willebrand factor A domain containing 3B	278
3:186281629–186282045	TBCCD1	TBCC domain containing 1	297
6:43112135–43112551	PTK7	PTK7 protein tyrosine kinase 7	306
14:64956734–64957150	ZBTB25	zinc finger and BTB domain containing 25	309
2:216239872–216240288	FN1	fibronectin 1	332
19:14217131–14217547	PRKACA	protein kinase, cAMP-dependent, catalytic, alpha	333
20:62903727–62904143	PCMTD2	protein-L-isoaspartate (D-aspartate) O-methyltransferase domain containing 2	385
14:32545502–32545918	ARHGAP5	Rho GTPase activating protein 5	390
2:24271847–24272263	C2orf44	chromosome 2 open reading frame 44	390
11:63754509–63754925	OTUB1	OTU domain, ubiquitin aldehyde binding 1	403
3:188665201–188665617	TPRG1	tumor protein p63 regulated 1	406
5:96294368–96294784	LNPEP	leucyl/cystinyl aminopeptidase	421
1:151694242–151694658	RIIAD1	regulatory subunit of type II PKA R-subunit (RIIa) domain containing 1	437
19:16204630–16205046	TPM4	tropomyosin 4	456
11:71163246–71163662	DHCR7	7-dehydrocholesterol reductase	460
12:3120506–3120922	TEAD4	TEA domain family member 4	504
1:200012019–200012435	NR5A2	nuclear receptor subfamily 5, group A, member 2	510
19:6007516–6007932	RFX2	regulatory factor X, 2 (influences HLA class II expression)	513
2:216300012–216300428	FN1	fibronectin 1	570
16:57474089–57474505	CIAPIN1	cytokine induced apoptosis inhibitor 1	590
3:42002666–42003082	ULK4	unc-51-like kinase 4 (C. elegans)	612
1:228644735–228645151	HIST3H2A	histone cluster 3, H2a	617
19:47735191–47735607	BBC3	BCL2 binding component 3	624
13:36919583–36919999	SPG20	spastic paraplegia 20 (Troyer syndrome)	629
22:30477422–30477838	HORMAD2	HORMA domain containing 2	630
2:235404366–235404782	ARL4C	ADP-ribosylation factor-like 4C	670
10:7860937–7861353	TAF3	TAF3 RNA polymerase II, TATA box binding protein (TBP)-associated factor, 140kDa	678
12:58160138–58160554	CYP27B1	cytochrome P450, family 27, subfamily B, polypeptide 1	688
6:35420636–35421052	FANCE	Fanconi anemia, complementation group E	706
19:13988601–13989017	NANOS3	nanos homolog 3 (Drosophila)	746
10:74870845–74871261	NUDT13	nudix (nucleoside diphosphate linked moiety X)-type motif 13	765
6:27115481–27115897	HIST1H2AH	histone cluster 1, H2ah	828
1:247266524–247266940	ZNF669	zinc finger protein 669	848
19:47216200–47216616	PRKD2	protein kinase D2	872
14:21946061–21946477	TOX4	TOX high mobility group box family member 4	886
19:11668857–11669273	ELOF1	elongation factor 1 homolog (S. cerevisiae)	895
17:36996603–36997019	C17orf98	chromosome 17 open reading frame 98	897
20:31123045–31123461	C20orf112	chromosome 20 open reading frame 112	947
12:50786921–50787337	LARP4	La ribonucleoprotein domain family, member 4	963
20:62340208–62340624	ZGPAT	zinc finger, CCCH-type with G patch domain	974
6:44188171–44188587	SLC29A1	solute carrier family 29 (nucleoside transporters), member 1	986
3:185001603–185002019	MAP3K13	mitogen-activated protein kinase kinase kinase 13	997
12:65089089–65089505	AC025262.1	Mesenchymal stem cell protein DSC96	1032
9:19050250–19050666	RRAGA	Ras-related GTP binding A	1086
16:1878116–1878532	FAHD1	fumarylacetoacetate hydrolase domain containing 1	1099
1:35323305–35323721	SMIM12	small integral membrane protein 12	1133
20:62461205–62461621	ZBTB46	zinc finger and BTB domain containing 46	1154
19:17531871–17532287	MVB12A	multivesicular body subunit 12A	1159
11:638254–638670	DRD4	dopamine receptor D4	1169
15:79576111–79576527	ANKRD34C	ankyrin repeat domain 34C	1173
3:55519903–55520319	WNT5A	wingless-type MMTV integration site family, member 5A	1220
7:148786349–148786765	ZNF786	zinc finger protein 786	1240
19:39437782–39438198	FBXO17	F-box protein 17	1253
19:47352074–47352490	AP2S1	adaptor-related protein complex 2, sigma 1 subunit	1291
2:201751795–201752211	PPIL3	peptidylprolyl isomerase (cyclophilin)-like 3	1299
12:92529288–92529704	RP11-24B21.1	uncharacterized protein LOC256021 isoform 1	1301
1:152007979–152008395	S100A11	S100 calcium binding protein A11	1324
13:103424546–103424962	TEX30	testis expressed 30	1351
6:107779175–107779591	PDSS2	prenyl (decaprenyl) diphosphate synthase, subunit 2	1377
1:31380013–31380429	SDC3	syndecan 3	1387
22:31004379–31004795	TCN2	transcobalamin II	1396
20:44423810–44424226	DNTTIP1	deoxynucleotidyltransferase, terminal, interacting protein 1	1414
19:46086441–46086857	OPA3	optic atrophy 3 (autosomal recessive, with chorea and spastic paraplegia)	1428
3:160115349–160115765	IFT80	intraflagellar transport 80 homolog (Chlamydomonas)	1438
14:23282743–23283159	SLC7A7	solute carrier family 7 (amino acid transporter light chain, y+L system), member 7	1440
18:686337–686753	ENOSF1	enolase superfamily member 1	1459
8:135650448–135650864	ZFAT	zinc finger and AT hook domain containing	1467

### Changes in 5hmC profile upon simulated-microgravity

**Fig 3:**
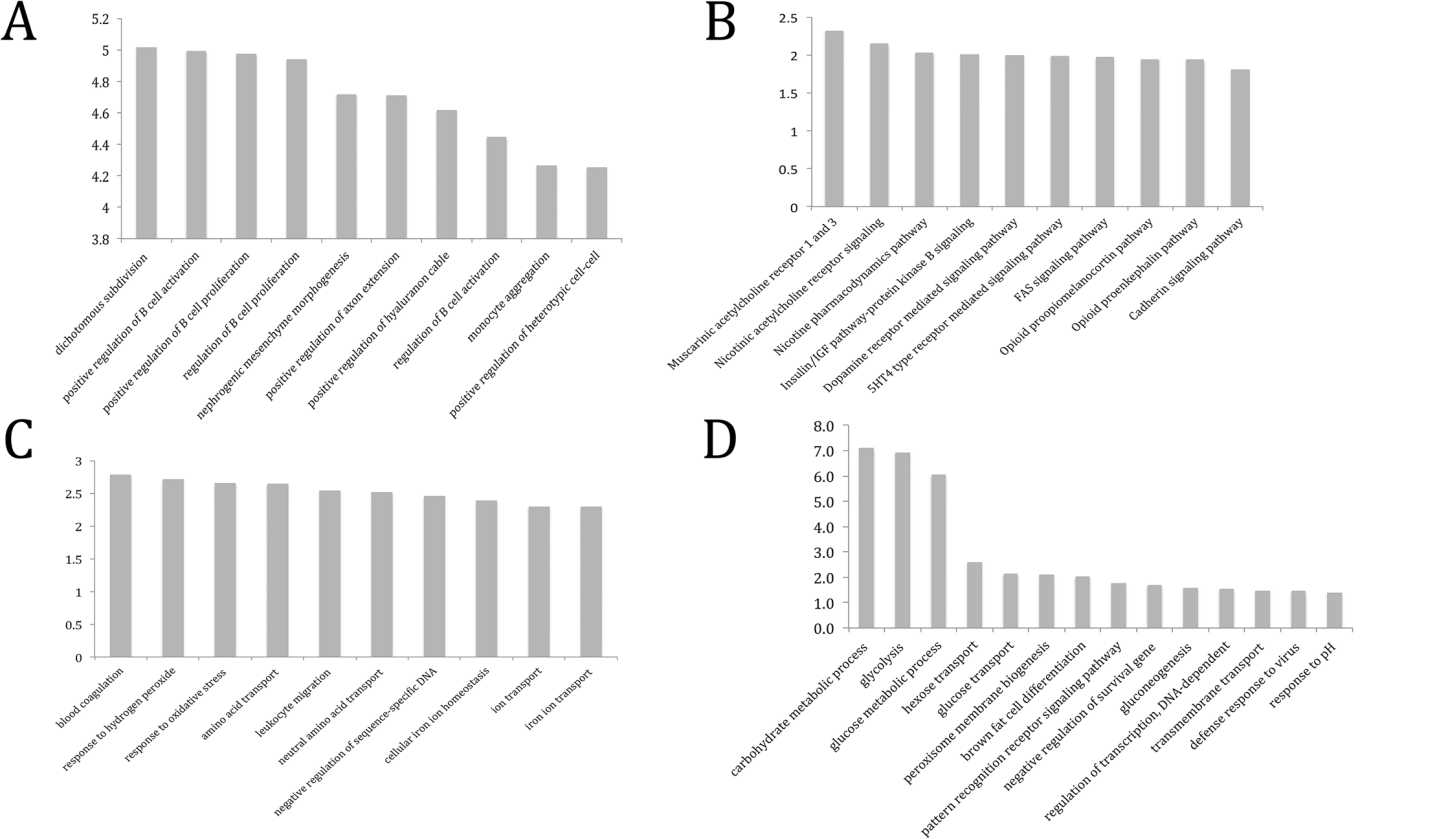
Pathways illustrating the network of genomic loci involved with (A & B) Regions undergoing increase in 5hmC content and (C & D) differential gene expression.

We applied hMeDIP analyses coupled with high-throughput sequencing to identify the differences in the genome-wide patterns of 5hmC upon simulated microgravity on TK6 cells. 2.7x10^8^ and 1.4x10^8^ reads were obtained during hMeDIP-seq from TK6 cells under static and simulated microgravity respectively and more than 90% of these read uniquely aligned to the human genome GRCh37/hg19, 2009 Assembly (**S1 Table and S2 Fig**). The depth of sequencing for the hmeDIP-seq samples ranged from 1.8X to 4.6X depending on the sample (**S1 Table**). Cross-correlation analysis was performed as per the ENCODE consortium guidelines [42, 52, 53] and all the samples displayed Normalized Strand Correlation (NSC) and Relative Strand Correlation (RSC) values greater than the minimum threshold (**S1 Table**). The consistency of reads in the biological replicates were observed through the cluster analysis (**S3 Fig**) and coverage analysis (**S5 Fig**). Of the 167 Differentially Hydroxymethylated Regions (DHMRs) **(S6 Table & S7 Table**) generated at IDR < 0.05, 154 (92.2%) were associated with hyper-hydroxymethylation (gain-of-5hmC) **(S6 Table)** and 13 (7.8%) with hypo-hydroxymethylation (loss-of-5hmC) (**S7 Table)** upon simulated microgravity respectively. The overlap of DHMRs with different genomic features indicated that 86 and 7 genes were associated with gain-of-5hmC and loss-of-5hmC DHMRs respectively (**Table 1**). Also, 5 gain-of-5hmC (**Table 4***)* and2 loss-of-5hmC (**Table 5)** DHMRs were observed around -1500 to 1500 bps of Transcription Start Sites (TSS). The distribution of DNA repeat regions present within the DHMRs was represented in **Table 1**. Metadata describing each DHMR was included in **S6 Table & S7 Table**. Investigation of GREAT version 3.0.0 ontology annotation [54] was included in **S8 Table.** Gain-of-5hmC DHMRs induced by simulated microgravity were found to be associated with genes that enriched in GO Biological Processes like positive regulation of B cell activation (GO: 0050871), positive regulation of B cell proliferation (GO: 0030890) and positive regulation of cell-cell adhesion (GO: 0034116) among others **(Fig 3A)**. Panther Pathway Analysis of these gan-of-5hmC DHMRs implicated the muscarinic acetylcholine receptor signaling (P00042), insulin/IGF pathway-protein kinase B signaling cascade (P00033) and Fas signaling (P00020) among others (**Fig 3B**), Due to an extremely small gene set associated with loss-of-5hmC DHMRs, significant p-values of pathway associations were not obtained and have not been reported here.

**Table 4:** List of hyper-hydroxymethylated DHMRs located within +/- 1500 of Transcription Start Sites of genes. Columns display the genomic coordinates of DHMRs, Gene Symbol of the corresponding gene, the description of the genome and the exact distance in base pairs.

DHMR(Chr:Start-End)	Gene Symbol	Description	Distance
7:73103920–73104390	WBSCR22	Williams Beuren syndrome chromosome region 22	-1091
19:11319910–11320380	DOCK6	dedicator of cytokinesis 6	-525
19:6678748–6679218	C3	complement component 3	229
20:29977639–29978109	DEFB119	defensin, beta 119	412
11:117745746–117746216	FXYD6	FXYD domain containing ion transport regulator 6	1359

**Table 5:** List of hypo-hydroxymethylated DHMRs located within +/- 1500 of Transcription Start Sites of genes. Columns display the genomic coordinates of DHMRs, Gene Symbol of the corresponding gene, the description of the genome and the exact distance in base pairs

DHMR(Chr:Start-End)	Gene Symbol	Description	Distance
3:96336458–96336934	MTRNR2L2	MT-RNR2-like 2	-579
3:96336159–96336635	MTRNR2L2	MT-RNR2-like 2	-280

### Changes in the transcriptome upon simulated-microgravity

In TK6 cells, simulated microgravity induced differential expression of 370 transcripts out of 22,376 transcripts analyzed (FDR<0.1) compared to static control **(S9 Table).** 271 (73.24%) differentially expressed transcripts were associated with a decrease in expression, while 99 (26.76%) differentially expressed transcripts were associated with an increase in gene expression. 17 (4.59%) genes were associated with a drastic change of differentially expression (greater than 2 fold increase or decrease), while the vast majority were associated with intermediate (0–2 fold) change in differential expression. Furthermore, the pathway analysis **(S10 Table)** of transcriptionally upregulated genes showed enrichment of GO Biological Processes such as response to oxidative stress (GO:0006979) and ion transport (GO:0006811) (**Fig 3C**), while the downregulated genes could be linked to regulation of DNA-dependent transcription (GO:0006355) and carbohydrate metabolic processes (GO:0005975) (**Fig 3D).** Some of the top upregulated genes include CHAC1, TRPA1, ATAD3C, INHBE, CTH, HMOX, HBD, SPG20, CACNA1D and PTGER4, while the top downregulated genes were GOLGA6L9, PFKFB4, FBXO17, ITGA6, PIK3R6, SLC2A5, INSIG2, AKAP6, HILPDA and POU2F3 (**S9 Table).**

### Correlation between simulated microgravity induced DMRS/DHMRs and gene expression

**Fig 4:**
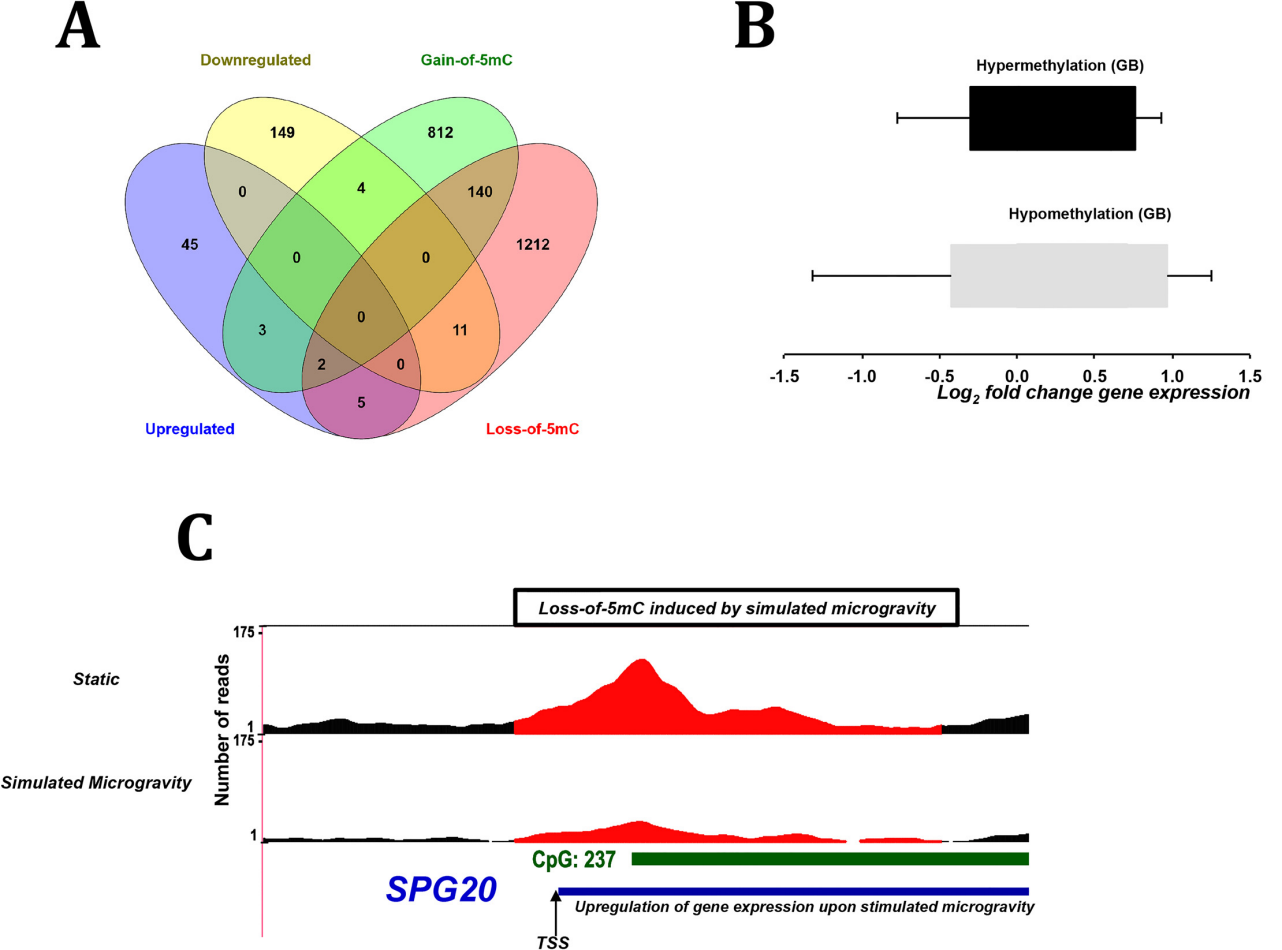
Overlap of simulated microgravity induced differentially expressed genes and genes undergoing differential methylation over gene-bodies (A) Fisher’s test showing no significant correlation between direction of methylation changes over gene bodies and their relative expression, (B)Venn diagram showing the overlap of differentially expressed genes with DMRs associated with gene bodies, (C) Statistical Representation of a gene SPG20, which underwent upregulation at the transcript level and a simultaneous decrease in 5mC levels at its promoter upon simulated microgravity.

A comparison of the simulated microgravity induced differentially expressed genes (**S9 Table**) with DMRs located at gene promoters (**Tables 2 & 3**) revealed that two transcriptionally upregulated genes (TSPAN5 and SPG20) were associated with loss-of-5mC at their promoter and three transcriptionally downregulated genes (PLIN2, MAP3K13 and FBXO1) were associated with loss-of-5mC at their promoter. None of the gene promoters linked to DHMRs (**Tables 4 & 5**) were found to be differentially expressed (**S9 Table**). Similarly, the comparison of simulated microgravity induced differentially expressed genes with DMRs/DHMRs located at gene bodies revealed that 25 differentially expressed associated with DMRs at their gene bodies and none of the differentially expressed genes associated with DHMRs at their gene bodies. The relationship between methylation status at gene bodies and their respective transcriptional activity of these 25 differentially expressed genes did not show any significant correlation by Fisher’s Exact Test (**Fig 4A**) and could be divided into five distinct groups, (i) five transcriptionally upregulated genes with loss-of-5mC DMRs at their gene bodies (CTH, CACNA1D, SPG20, PLS1 and SLC39A14), (ii) eleven transcriptionally downregulated genes with loss-of-5mC DMRs at their gene bodies (FBXO17, AKAP6, RIT1, GTF2IRD2P1, MSTO1, PMS2CL, MAP3K13, ST3GAL1, NCKIPSD, MAST1 and MSTO2P), (iii) three transcriptionally downregulated genes associated with gain-of-5mC DMRs at their gene bodies (CACNB2, WDR45B and CABLES1), (iv) three transcriptionally upregulated genes with gain-of-5mC DMRs at their gene bodies (CASZ1, VCL and ATF3) and (v) two transcriptionally upregulated genes with gain-of-5mC as well as loss-of-5mC DMRs at their gene bodies (ARID5B and TSPAN5) (**Fig 4B**). The comparison of DMRs and DHMRs located at gene bodies (**S6 Fig**) yielded six overlapping groups namely (i) 140 genes were associated with gain-of-5mC and loss-of-5mC DMRs at their gene bodies, (ii) eight were genes were associated with loss-of-5mC DMRs and gain-of-5hmC DHMRs, (iii) five gene were associated with gain-of-5mC and loss-of-5mC DMRs as well as gain-of-5hmC DHMRs at their gene bodies, (iv) seven genes were associated with gain-of-5mC DMRs and gain-of-5hmC DHMRs at their gene bodies, (v) one gene was associated with gain-of-5mC DMR and loss-of-5hmC DHMR at its gene body and (vi) two genes were associated with gain-of-5hmC DHMRs and loss-of-5hmC DHMRs at their gene bodies.

## Discussion

The objective of this ground-based study was to map the genome-wide effects of simulated microgravity on DNA methylation, hydroxymethylation; and gene expression patterns in TK6 lymphoblastoid cells by a powerful Next Generation Sequencing pipeline. Although on the basis of numerous studies reporting microgravity-induced physiological changes in living organisms ranging from prokaryotes to humans, it has been speculated that microgravity-induced changes may occur in the methylome, very little is known about the effects of microgravity on DNA methylation. In 2009, Ou *et al* reported hypermethylation of a set of genes and transposable elements in rice (*Oryza sativa L*.*)* plants germinating from space-flown seeds [29]. Ou *et al* also reported that the spaceflight-induced hypermethylated genes did not generally correlate with alterations in their gene expression status [29]. In 2010, Singh *et al* reported that human T-lymphocytes subjected to simulated microgravity underwent global DNA hypomethylation on the basis of Methylation Sensitive-Random Amplified Polymorphic DNA (MS-RAPD)-PCR analysis [30]. However, since MS-RAPD-PCR is unable to identify specific methylated sites, the study by Singh *et al* could not report the target genes associated with the simulated-microgravity induced DNA hypomethylation. In 2011, Ou *et al* validated their previous finding that spaceflight induced hypermethylation of DNA (the frequency of spaceflight-induced hypermethylation was demonstrated to be nearly double of spaceflight-induced hypomethylation events) by assessing a larger genomic subset comprising 467 loci [31], though it was not evident if any study to correlate changes in DNA methylation with gene expression were further conducted.

The disparity between the conclusions of the studies conducted by Ou *et al* and Singh *et al* could be attributed to several factors, but we think that the following might be important to consider: (i) differences in mechanisms establishing and maintaining DNA methylation patterns in plants and animals (for a comprehensive review refer to [55]), (ii) while Ou *et al*’s investigation was based on spaceflight-induced “epigenetic memory” being transmitted from the seeds to the sapling, Singh *et al* had investigated the simulated microgravity-influenced changes in DNA methylation in immortalized T-lymphocyte cell cultures that might not be inheritable and (iii) while Singh *et al* had investigated the effects of only simulated microgravity on DNA methylation, Ou *et al* was investigating the effects of numerous factors like cosmic radiation, microgravity and space magnetic fields encountered during spaceflight. These reports therefore provided a strong basis for us to perform this study with advanced methods such as MeDIP-seq, hMeDIP-seq and RNA-seq to explore the relationship between the methylome and the transcriptome in microgravity exposed cells. To the best of our knowledge, this is the first report profiling the effects of simulated microgravity on the epigenomic landscape of human cells. 3204 DMRs and 2116 DHMRs distributed throughout the genome were identified in TK6 cells subjected to simulated microgravity. The majority of the DMRs (59.86%) were identified to undergo hypomethylation, which was consistent with the findings of Singh *et al* [30]. On the other hand the majority of DHMRs (92.2%) were associated with hyper hydroxymethylation.

Additionally, we have been able to perform ontology based annotations to obtain information about the biological processes that might be affected by genes associated with simulated microgravity induced changes occurring in the methylome. In particular, genes involved in primary metabolic processes, immune functions and the p53 pathway seems to be undergoing changes in their methylation/hydroxymethylation status under the influence of simulated microgravity. An early study on lymphoblastoid cells subjected to 48 hours of simulated microgravity by Degan *et al*. reported decrease of cellular ATP content, suggesting a simulated microgravity induced alteration in cellular metabolism [56]. It remains to be seen how simulated microgravity induced changes over the methylation levels of p53 effector genes play in TK6 which expresses the wild-type p53 [57], a tumor suppressor functioning extensively in the DNA repair pathway. Reduction of global methylation has been proposed to be a hallmark of genomic instability [14, 58] and it remains to be seen if the extensive loss-of-5mC induced by simulated microgravity reported in this study has any functional implications. Another finding in TK6 cell line, which was originally derived from a patient with T- blast crisis [32, 59, 60] and could potentially harbor progenitor forms of lymphocytes, pertains to changes in methylation/hydroxymethylation patterns over genes involved in lymphocyte development and activation cultured under simulated microgravity conditions. Interestingly whole-exome sequencing has revealed similarities in the genomic content of lymphocytes and lymphoblastoid cells [61], and thus in light of our findings TK6 lymphoblastoid cells may emerge as a good model to study B and T- lymphocyte development and activation in *in vitro* genomic studies.

Our study also revealed that simulated microgravity could alter the expression of 370 transcripts, however only 17 of these underwent greater than 2-fold change of up/downregulation. The transcriptionally upregulated genes showed enrichment of pathways involving response to oxidative stress and negative regulation of gene expression, while the downregulated genes could be linked to pathways responsible for glucose metabolism and transcription regulation. While our study illustrated that there was no direct relationship between differentially expressed genes and changes in 5mC/5hmC over its promoters/gene bodies, we have been able to determine the methylation status of individual genes implicated in earlier studies to be affected in transcriptional or translational activities on exposure to simulated microgravity in ground based studies or in spaceflights. For instance, the voltage-dependent calcium channel L-type, alpha 1D (CACNA1D) gene transcript was observed to be differentially expressed in human T-lymphocytes subjected to microgravity conditions during spaceflight compared to ground static controls [49]. While, we observed a nominal increase at the transcript level for CACNA1D, we observed a decrease in 5mC levels over its gene body under simulated microgravity.

In another study, the Activating Transcription Factor-3 (ATF3) has been implicated to be differentially expressed upon being subjected to microgravity during spaceflight in cultured HUVEC cells [62]. Our RNA-seq data illustrated an increase of 1.3 fold in the transcript level of ATF3 and a decrease in the 5mC levels over its gene body under the influence of simulated microgravity. Interestingly, ATF3, a member of the ATF/CREB family of transcription factors, has been observed to be upregulated when cells are exposed to stress conditions [50]. On the other hand, Integrin alpha-6 (ITGA6) which is an integral cell surface protein has been observed to be down-regulated at the transcriptional scale during short-term weightlessness produced by parabolic maneuvers in human cells [51]. While RNA-seq revealed a decrease of ITGA6 transcript by more than 2-fold, we were not able to observe changes in the 5mC and 5hmC profile over its gene body or promoter, implying that possibly mechanisms other than DNA methylation might be involved in its regulation.

Some of the novel gene functions that we have linked with DNA methylation status include the F-Box Protein 17 (FBXO17), which constitutes one of the four subunits of the ubiquitin-protein-ligase complex called SKP1-cullin-F-box (SCFs) and mediates substrate specificity [63, 64]. While the transcript level of FBXO17 was observed to be downregulated by 2.47 fold, the 5mC levels over the gene body of FBXO17 (chr19:39437782–39438198) decreased in TK6 cells subjected to simulated microgravity. Recently it has been demonstrated that the recruitment of F-box motif bearing homologous protein in yeast Met30 is regulated by a complex mechanism and has been implicated in stress response [65, 66]. In sync with these observations, reduction of 5mC levels over gene bodies of other F-Box motif containing proteins such as FBXO31 (chr16:87421262–87421678) and FBXO42 (chr1:16674945–16675361) and promoter of FBXO5 (2024 bps upstream of TSS; chr6:153306530–153306946), was also observed though their transcripts were not differentially expressed. Interestingly, genes which function as molecular mechano-sensors like Vinculin (VCL) or mediate stress-signal transduction events like Tetraspan-5 (TSPAN5) were also seen to undergo changes in its gene methylation levels and expression. Similarly, other genes involved in the Metabolic process (GO:0008152) like Cystathionine gamma-lyase (CTH), Phospholipid scramblase-1 (PLS1) Microtubule-associated serine/threonine-protein kinase-1 (MAST1), Zinc finger protein castor homolog-1 (CASZ1), CMP-N-acetylneuraminate-beta-galactosamide-alpha-2,3-sialyltransferase-1 (ST3GAL1), AT-rich interactive domain-containing protein-5B (ARID5B), Mitogen-activated protein kinase-13 (MAP3K13) and Perilipin-2 (PLIN2) were implicated in this study contributing to mechano-stress response. Though our study does not show a global correlation between methylation status and transcriptional activity, the simulated microgravity induced changes over SPG20 (a gene implicated in endosomal trafficking and mitochondrial functions) recapitulates the conventional theory of decrease in promoter methylation corresponding to elevated gene activity. This novel finding suggests that methylation-dependent transcriptional activity is not a genome-wide phenomenon, instead it may be applicable for specific genes.

Thus, in conclusion we believe that 48 hours of treatment with simulated microgravity triggered changes in the transcriptome particularly involving biological processes such as negative regulation of transcription, response to stress and reduction in carbohydrate metabolic processes. This study revealed that simulated microgravity influenced alteration of genome-wide 5mC and 5hmC patterns, however no correlation was found between DMRs/DHMRs situated at gene bodies and promoters and their transcriptional status. While it has been long held that genes with methylated promoters are transcriptionally silent, recent studies have uncovered the association of methylated gene promoters with both transcriptionally active and inactive genes [20, 21, 67–70]. On the other hand, gene body methylation has been observed to be positively correlated with gene expression in some studies [71, 72] and no such correlation has been found in others [22, 73–75]. Recent deep-sequencing based explorations have challenged the traditional paradigm and illustrated complexities of the nature of relationship between DNA methylation and gene expression [19–25]. It is also conceivable that pronounced alterations in epigenetic patterns may take place in cells subjected to prolonged microgravity environments. The ground-based microgravity simulators like the one used in our study have undoubtedly enhanced our understanding of microgravity but it has to be pointed out that the principle of “simulating” microgravity involves changing the direction of Earth’s gravity subjected to the samples through continuous rotation and represent “functional near weightlessness”. While this is the first study to profile the simulated microgravity induced changes in 5mC/5hmC patterns and gene expression simultaneously providing a perspective of epigenetic alterations we could expect during short-term exposures, our understanding is far from complete. We believe that genes involved in altered biological processes identified in this study will be of considerable interest and provide a valuable resource for future investigations. Finally, in the interest of astronauts who are exposed to microgravity for prolonged periods of time, future studies should focus on performing time course experiments monitoring the influence of “real” and “simulated” microgravity exposure on a variety of models to determine the precise effects of microgravity on the epigenome

## Supporting Information

S1 Fig TK6 cell count under simulated microgravity (12 rpm) and static (control) conditions in the replicates. (TIF)

S2 Fig Sequencing Summary. The total number of reads (white) and the total number of unique reads aligned to the human genome (blue) obtained by performing hmeDIP-seq and meDIP-seq on TK6 cells cultured under static (control) and simulated microgravity (12 rpm) conditions for 48 hours. S1 Table demonstrates the exact numbers and percentage of mapped reads.
(TIF)

S3 Fig Cluster analysis performed on the reads obtained on meDIP-seq and hmeDIP-seq on TK6 cells cultured under static (control) and simulated microgravity conditions which shows similarities in the biological replicates of each condition. (TIF)

S4 Fig Coverage analyses performed in MeDUSA using the MEDIPS bioconductor package on the reads generated from (A) MeDIP-seq on control replicate A, (B) MeDIP-seq on control replicate B, (C) MeDIP-seq on simulated microgravity exposed replicate A and (D) MeDIP-seq on simulated microgravity exposed replicate B, over 28217009 CpG dinucleotides. Color of these lines represent the fold coverage of the CpGs as shown in the legend.
(TIF)

S5 Fig Coverage analyses performed in MeDUSA using the MEDIPS bioconductor package on the reads generated from (A) hMeDIP-seq on control replicate A, (B) hMeDIP-seq on control replicate B, (C) hMeDIP-seq on simulated microgravity exposed replicate A and (D) hMeDIP-seq on simulated microgravity exposed replicate B, over 28217009 CpG dinucleotides. Color of these lines represent the fold coverage of the CpGs as shown in the legend.
(TIF)

S6 Fig Venn diagram showing overlap of genes whose gene body was found to be associated with DMRs and DHMRs (gain-of-5mC/hmC and loss-of-5mC/hmC) (TIF)

S1 Table Sequencing summary quality statistics. (XLSX)

S2 Table List of simulated microgravity-induced DMRs undergoing hypermethylation. For every DMR identified, a description of the genomic features found in this region has been provided. The columns represent the following information: (A) Genomic coordinates of the region defined as a DMR and (B) ENCODE IDs of features (such as gene, transcript, pseudogene, non-coding RNA or other regulatory feature) present in the region.
(XLSX)

S3 Table List of simulated microgravity-induced DMRs undergoing hypomethylation. For every DMR identified, a description of the genomic features found in this region has been provided. The columns represent the following information: (A) Genomic coordinates of the region defined as a DMR and (B) ENCODE IDs of features (such as gene, transcript, pseudogene, non-coding RNA or other regulatory feature) present in the region.
(XLSX)

S4 Table GREAT Ontology Summary Statistics for hypermethylated DMRs. The columns represents the respective ontology name, term name / identifier, term description, binomial rank, binomial p-value (uncorrected), binomial Bonferroni corrected p-value, binomial FDR q-value and names of gene hits generated by GREAT version 3.0.0; Species assembly: hg19 and association rule: Basal+extension: 5000 bp upstream, 1000 bp downstream, 1000000 bp max extension, curated regulatory domains included.
(XLSX)

S5 Table GREAT Ontology Summary Statistics for hypomethylated DMRs. The columns represents the respective ontology name, term name / identifier, term description, binomial rank, binomial p-value (uncorrected), binomial Bonferroni corrected p-value, binomial FDR q-value and names of gene hits generated by GREAT version 3.0.0; Species assembly: hg19 and association rule: Basal+extension: 5000 bp upstream, 1000 bp downstream, 1000000 bp max extension, curated regulatory domains included.
(XLSX)

S6 Table List of simulated microgravity-induced DHMRs undergoing hyper-hydroxymethylation. For every DHMR identified, a description of the genomic features found in this region has been provided. The columns represent the following information: (A) Genomic coordinates of the region defined as a DHMR and (B) ENCODE IDs of features (such as gene, transcript, pseudogene, non-coding RNA or other regulatory feature) present in the region.
(XLSX)

S7 Table List of simulated microgravity-induced DHMRs undergoing hypo-hydroxymethylation. The columns represent the following information for each identified DHMR: (A) Genomic coordinates of the region defined as a DHMR and (B) ENCODE IDs of features (such as gene, transcript, pseudogene, non-coding RNA or other regulatory feature) present in the region.
(XLSX)

S8 Table GREAT Ontology Summary Statistics for hyperhydroxymethylated DHMRs. The columns represents the respective ontology name, term name / identifier, term description, binomial rank, binomial p-value (uncorrected), binomial Bonferroni corrected p-value, binomial FDR q-value and names of gene hits generated by GREAT version 3.0.0; Species assembly: hg19 and association rule: Basal+extension: 5000 bp upstream, 1000 bp downstream, 1000000 bp max extension, curated regulatory domains included.
(XLSX)

S9 Table List of Differentially Expressed Genes induced by simulated microgravity. The columns represent geneID, name of gene from UCSC Genome Browser (duplicates exist because multiple geneID can map to same gene), chromosome location, strand: + or–, transcription start position, transcription end position, the log2-fold-change of gene expression, the average log2-counts-per-million of comparison, p-value of comparison, false discovery rate (corrected p-value) of comparison.
(XLSX)

S10 Table Pathway Analysis of simulated microgravity induced differentially up and downregulated genes. (XLSX)
