# Supplementary figures and images for: A Study of Alterations in DNA Epigenetic Modifications (5mC and 5hmC) and Gene Expression Influenced by Simulated Microgravity in Human Lymphoblastoid Cells
Source: PLoS One. 2016 Jan 28;11(1):e0147514. doi: 10.1371/journal.pone.0147514 (PMC4731572; doi:10.1371/journal.pone.0147514)

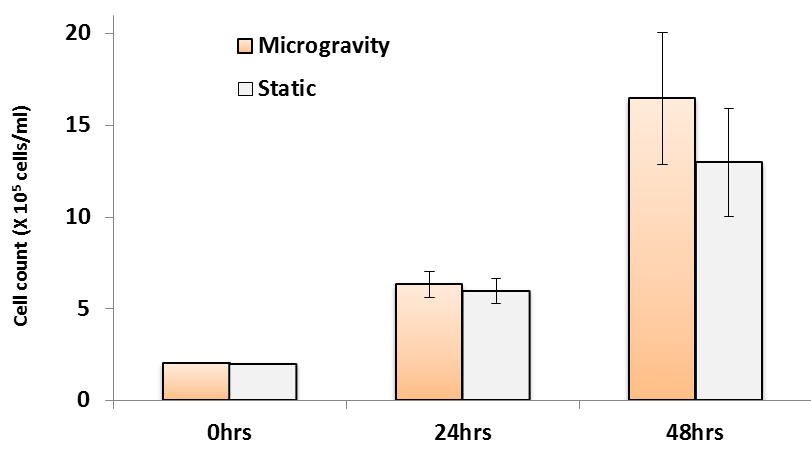

Supplement: S1 Fig — (TIF) [file pone.0147514.s001.tif]

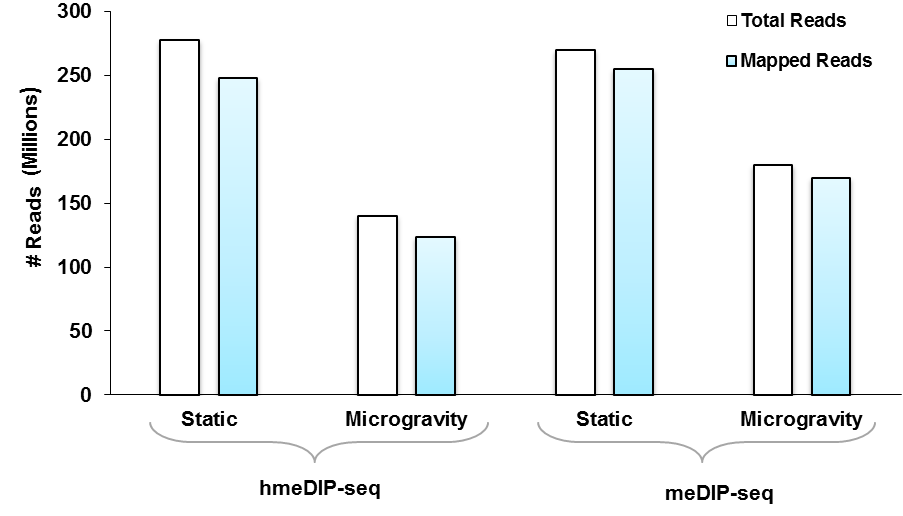

Supplement: S2 Fig — The total number of reads (white) and the total number of unique reads aligned to the human genome (blue) obtained by performing hmeDIP-seq and meDIP-seq on TK6 cells cultured under static (control) and simulated microgravity (12 rpm) conditions for 48 hours. S1 Table demonstrates the exact numbers and percentage of mapped reads. (TIF) [file pone.0147514.s002.tif]

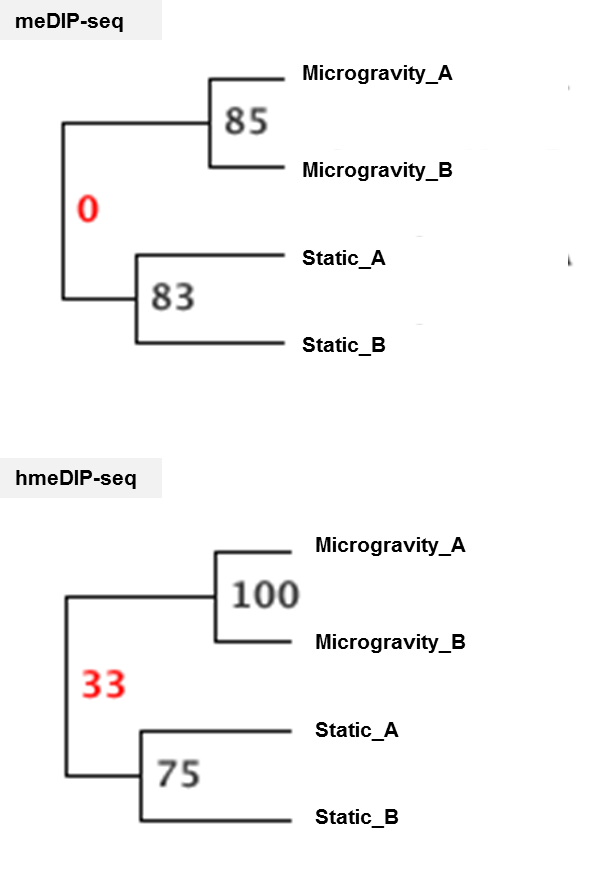

Supplement: S3 Fig — (TIF) [file pone.0147514.s003.tif]

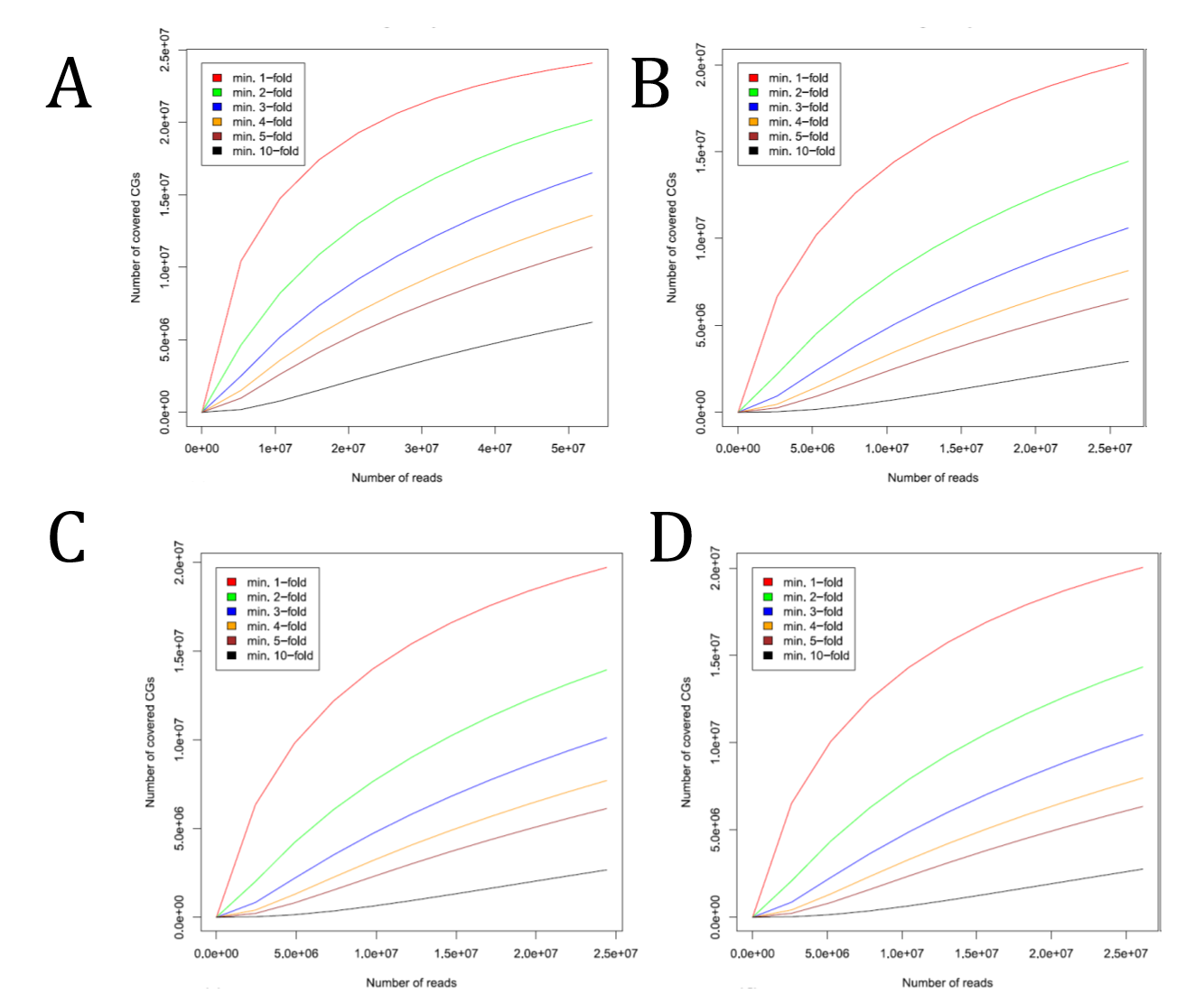

Supplement: S4 Fig — Color of these lines represent the fold coverage of the CpGs as shown in the legend. (TIF) [file pone.0147514.s004.tif]

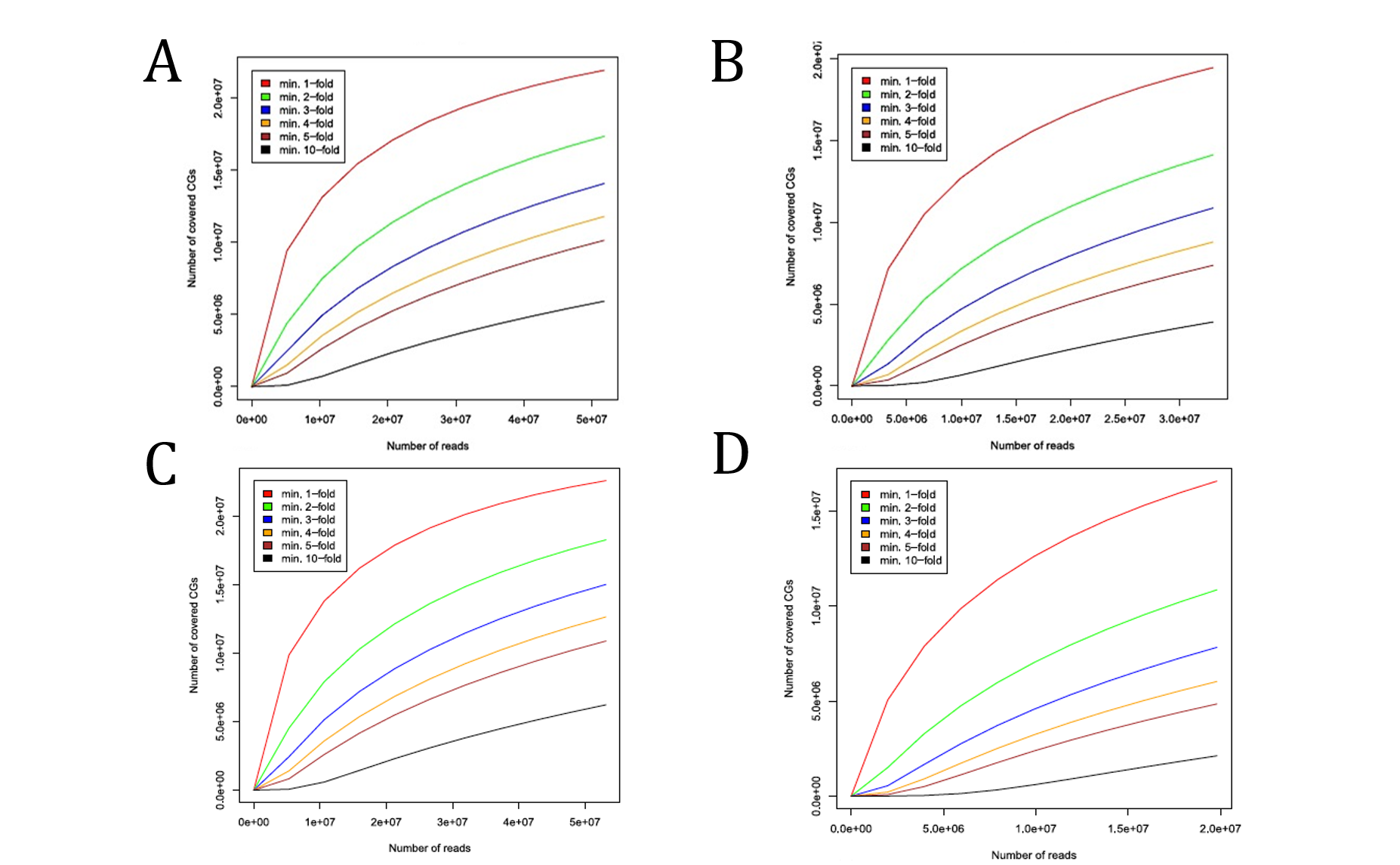

Supplement: S5 Fig — Color of these lines represent the fold coverage of the CpGs as shown in the legend. (TIF) [file pone.0147514.s005.tif]

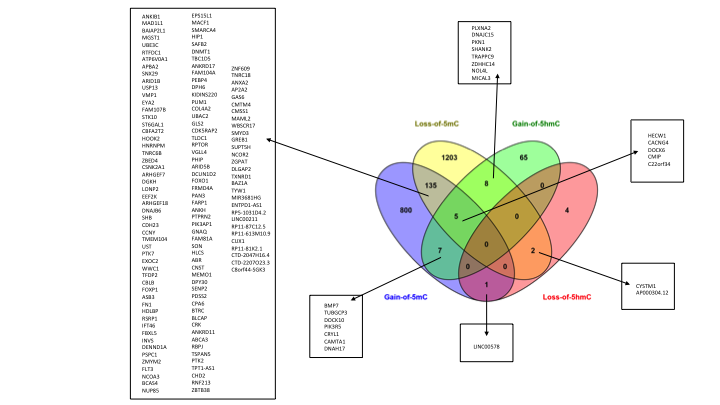

Supplement: S6 Fig — (TIF) [file pone.0147514.s006.tif]
